# Development and validation of an instrument for nursing consultation with pediatric patients in the preoperative period^*^

**DOI:** 10.1590/1980-220X-REEUSP-2021-0467

**Published:** 2022-03-28

**Authors:** Paloma Gonçalves Martins Acioly, Eny Dórea Paiva, Adriana Teixeira Reis, Tatiana de Oliveira Gomes, Luciana Rodrigues da Silva, Liliane Faria da Silva

**Affiliations:** 1 Fundação Oswaldo Cruz, Instituto Nacional de Saúde da Mulher, da Criança e do Adolescente Fernandes Figueira, Rio de Janeiro, RJ, Brazil.; 2 Universidade Federal Fluminense, Escola de Enfermagem Aurora Afonso Costa, Niterói, RJ, Brazil.; 3 Universidade do Estado do Rio de Janeiro, Faculdade de Enfermagem, Rio de Janeiro, RJ, Brazil.

**Keywords:** Pediatric Nursing, Perioperative Care, Nursing Process, Validation Study, Standardized Nursing Terminology, Nursing Assessment, Enfermeria Pediátrica, Atención Perioperativa, Proceso de Enfermeria, Estudio de Validación, Terminologia Normalizada de Enfermeria, Evaluación em Enfermeria, Enfermagem Pediátrica, Assistência Perioperatória, Processo de Enfermagem, Estudo de Validação, Terminologia Padronizada em Enfermagem, Avaliação em Enfermagem

## Abstract

**Objective:**

To develop and validate an instrument for nursing consultation with pediatric patients in the preoperative period.

**Method:**

This is a methodological study, consisting of five steps: identification of nursing diagnoses, discussion and evaluation of diagnoses with nurses from the institution, instrument development, instrument content validation with *experts* through the Delphi Technique, and instrument restructuring. The Nursing Minimum Data Set, Wanda Horta’s human needs, and the NANDA-NOC-NIC connections were used as theoretical framework.

**Results:**

In its final version, the instrument includes an assessment of psychobiological, psychosocial, and psychospiritual human needs, 38 nursing diagnoses, 65 nursing interventions, 113 nursing activities, and 62 nursing outcomes. The instrument obtained a content validity index between 0.90 and 1.0 in the first round, and suggestions, validated in the second round, obtained agreement from 70 to 100%.

**Conclusion:**

The instrument developed can be a tool for use in nursing consultations in the preoperative period for children, providing greater assertiveness to nursing actions for this clientele.

## RESUMO

**Objetivo:**

Elaborar e validar um instrumento para consulta de enfermagem ao paciente pediátrico em pré-operatório.

**Método:**

Estudo metodológico, composto por cinco etapas: identificação dos diagnósticos de enfermagem, discussão e avaliação dos diagnósticos com enfermeiros da instituição, elaboração do instrumento, validação do conteúdo do instrumento com *experts* pela Técnica Delphi e reestruturação do instrumento. Foram utilizados o Conjunto de Dados Mínimos de Enfermagem, as necessidades humanas de Wanda Horta e as ligações NANDA-NOC-NIC como referencial teórico.

**Resultados:**

Em sua versão final, o instrumento contempla avaliação das necessidades humanas psicobiológicas, psicossociais e psicoespirituais, 38 diagnósticos de enfermagem, 65 intervenções, 113 atividades e 62 resultados de enfermagem. O instrumento obteve índice de validade de conteúdo entre 0,90 e 1,0 na primeira rodada, e sugestões, validadas na segunda rodada, obtendo concordância de 70 a 100%.

**Conclusão:**

O instrumento elaborado pode ser uma ferramenta para uso em consulta de enfermagem no pré-operatório infantil, conferindo maior assertividade nas ações de enfermagem a essa clientela.

## RESUMEN

**Objetivo:**

Elaborar y validar un instrumento para consulta de enfermería al paciente pediátrico en preoperatorio.

**Método:**

Estudio metodológico, compuesto por cinco etapas: identificación de los diagnósticos de enfermería, discusión y evaluación de los diagnósticos con enfermeros de la institución, elaboración del instrumento, validez del contenido del instrumento con expertos por la Técnica Delphi y reestructuración del instrumento. Se utilizaron el Conjunto de Datos Mínimos de Enfermería, las necesidades humanas de Wanda Horta y la nomenclatura NANDA-NOC-NIC.

**Resultados:**

En su versión final, el instrumento contempla evaluación de las necesidades humanas psicobiológicas, psicosociales y psicoespirituales, 38 diagnósticos de enfermería, 65 intervenciones, 113 actividades y 62 resultados de enfermería. El instrumento obtuvo índice de validez de contenido entre 0,90 y 1,0 70 en la primera etapa, y sugerencias, validadas en la segunda etapa que obtuvieron concordancia de 70 a 100%.

**Conclusión:**

El instrumento elaborado puede ser una herramienta para uso en consulta de enfermería en el preoperatorio infantil, proporcionando más seguridad en las acciones de enfermería a ese público.

## INTRODUCTION

The imminence of a surgical intervention inserts new situations and sensations into the family routine, which can be extremely stressful and impactful for children and their families^(1,2)^.

The nurse shall understand the importance of a surgical procedure for the patient and intercede with appropriate strategies for the age and existing pathologies^(3)^. In the preoperative period, Nursing interventions can help the patient and his/her family reducing anxiety, providing for better recovery and greater patient satisfaction with the care received^(3)^.

Nursing assistance and guidance during the preoperative period can avoid surgery postponement and unfavorable consequences for the patient, his/her family, the institution, and the health team involved^(4,5,6)^.

When considering the surgical patient’s specific characteristics, studies emphasize the importance of efforts to improve the quality of nursing care^(7,8)^. In this regard, this quality can be achieved through the use of the Nursing Process (NP), which organizes care in three ways: methodologically, personally, and instrumentally^(7)^.

Resolution 358/2009 of the Brazilian Federal Nursing Council (COFEN) establishes the Systematization of Nursing Care (SAE) and the NP^(9)^. The NP, when performed in institutions providing outpatient health services, is called nursing consultation^(10)^.

To classify and name all the events, diagnoses, interventions, and outcomes included in the development of the NP, the classifications of the taxonomy of the North American Association of Nursing Diagnosis – NANDA, the Classification of Nursing Interventions – NIC, and the outcomes proposed in the Nursing Outcomes Classification – NOC, references for Brazilian and international nursing^(11)^, were selected.

The nursing diagnoses and interventions found in the literature are comprehensive, and their use is limited specifically for the child population^(4,12)^.

The lack of instruments that can guide the preoperative nursing consultation and the scarcity of literature covering nursing diagnoses and specific nursing interventions for the pediatric surgical patient were motivators for this research, which aimed to develop and validate an instrument of nursing consultation to the pediatric patient in the preoperative period.

## METHOD

### Design of Study

This is a methodological study, performed from January 2019 to August 2020, which was divided in five stages: identification of nursing diagnoses, discussion and evaluation of diagnoses with nurses from the institution, instrument development, instrument content validation with experts through the Delphi Technique, and instrument restructuring.

### Stage 1 – Identification of Nursing Diagnoses

An integrative review was carried out, in the data sources *Medical Literature Analysis and Retrieval System Online* (MEDLINE), *Latin American and Caribbean Literature in Health Sciences* (LILACS), Brazilian Nursing Database – Brazilian Bibliography (BDENF), and in the platform *Cumulative Index to Nursing and Allied Health Literature* (CINAHL), aiming to identify the possible nursing diagnoses for the pediatric patient in the preoperative period, using the descriptors “*Nursing Diagnosis*” and “P*ediatrics*” in Portuguese, English and Spanish, published from 2015 to 2019. The inclusion criteria were studies addressing the topic “nursing diagnoses for pediatric patients”, published from 2015 to 2019. As exclusion criteria, the following were outlined: being a dissertation or thesis, previous note, expert opinion, and all articles that did not answer the review question. Of a total of 157 articles found in the review, 03 were excluded due to duplication, 154 were screened, 133 of which were excluded after title and abstract reading, and 21 were considered for analysis; of these, 09 were excluded after full reading, and 12 articles relevant to the research remained. The twelve articles selected were analyzed and organized for data collection, according to the review protocol. Thus, 89 possible nursing diagnoses were listed for the pediatric patient in the preoperative period.

### Stage 2 – Discussion and Evaluation of Diagnoses with Nurses

In the second stage, the 89 possible nursing diagnoses listed by the integrative review were grouped, with similarities between them being checked and related to the NANDA I 2018-2020 taxonomy diagnoses^(12)^. Forty-nine nursing diagnoses remained for discussion at the first meeting with nurses. Discussion and evaluation of diagnoses took place at the institution, where the research was carried out, through two meetings with local nurses.

Six nurses attended the meeting: two with a doctorate degree, three with a master’s degree, and one with a graduate certificate, all of them in Pediatrics. All had experience in pediatric surgical patient care, and five of them had more than 10 years of experience.

The condensed information with 35 diagnoses discussed and approved at the first meeting and 07 suggested by the researcher, totaling 42 nursing diagnoses, was sent via email for approval by the nurses. Moreover, the dialogs took place virtually, through the application *Whatsapp,* due to the COVID-19 pandemic. The 38 nursing diagnoses approved in the meetings were used in the elaboration of the instrument, being later validated by the experts.

### Stage 3 – Instrument Development

In the third stage, the Nursing Minimum Data Set – NMDS^(13)^ was used considering the structure and the initial part of the instrument where the patient and institution identification data are, with some modifications: the “Admission date” was replaced by the “Date of consultation” and the “Date of discharge” by “Date of surgery”^(13)^.

For the elaboration of the part of the instrument related to the collection of patient data, the framework of Wanda Horta’s Human Needs was used with some adjustments by Garcia and Cubas^(14)^, aiming at identifying the pediatric patient’s needs in the preoperative period. Nursing planning and care were supported by the 38 diagnoses approved in the first stages and the nursing interventions and outcomes correlated and mapped with the NANDA-NIC-NOC links^(11)^.

### Stage 4 – Instrument Validation

In the fourth stage, the instrument content was validated using the Delphi method^(15)^. The experts were intentionally selected according to their experience and knowledge related to the study topic, following Fehring’s criteria^(16)^. The search for them was made consulting the curricula available on CAPES’s (Coordination for the Improvement of Higher Education Personnel) Lattes Platform or by indication of specialists.

The experts carried out the validation considering the relevance of data related to the institution, patient identification data, as well as data to be collected in the assessment of the human needs of the pediatric surgical patient and related to the planning of nursing care.

### Stage 5 – Instrument Restructuring

In the fifth stage, the instrument was restructured based on the items validated after the rounds with the experts.

### Data Collection

Invitations to participate were sent via email or via *Whatsapp,* to 35 specialists from all regions of Brazil, which contained a link to access Google Forms, and which was responded anonymously. Ten experts accepted participating.

After the experts filled the form out, data collected were returned by e-mail. For the instrument content validation, the degree of relevance of each item was analyzed through the following answer alternatives: 4 – extremely relevant, when the expert considered the item’s relationship with the altered/affected human need and with the assessment and planning of nursing care to be very important; 3 – relevant, when he/she considered it important; 2 – little relevant, the item has little importance; and 1 – irrelevant, the item was not considered important regarding patient data and nursing care planning^(17)^.

### Data Analysis and Treatment

Instrument items validation by the *experts* was evaluated by the Content Validity Index (CVI), which allowed the analysis of the items individually using *Likert*-type scale with a score of one to four^(17)^. This was calculated through the sum of agreement of the items that obtained alternatives 3 and 4 from the specialists. In this study, items with at least 0.7/70% agreement were considered valid. For the evaluation of each item, the formula below was used^(17)^:

CVI=number of responses 3 or 4/total number of responses

A final round was required to evaluate suggestions and inclusions. These were considered validated when they obtained 70% agreement among the experts. For the analysis of the results, the Content Index (CI) was used^(17)^. This method is used to calculate the percentage of agreement among the specialists according to the formula below^(17)^:

% agreement=number of participants who agreed/ total number of participants × 100

Once the results were obtained with the application of these indices, the validation process was terminated.

### Ethical Aspects

The present study was carried out in accordance with the norms of Resolution no. 466, of December 12, 2012, of the Brazilian Health Council and was approved by the Participating Institution (Opinion No. 3.627.393 of 10/08/2019) and by the Co-Participating Institution (Opinion No. 3.785.239 of 12/19/2019).

## RESULTS

**Figure 1.**
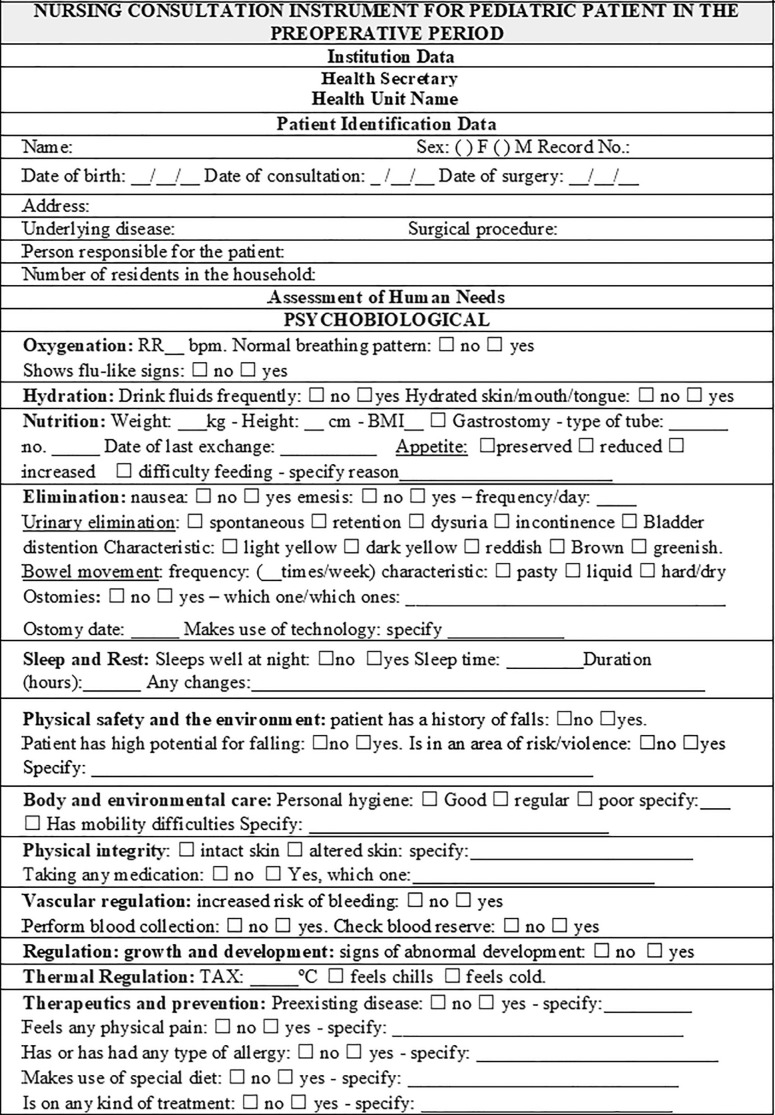
Final Version of the Instrument – Identification and Assessment of Human Needs: Psychobiological. Niterói, RJ, Brazil, 2020.

**Figure 2.**
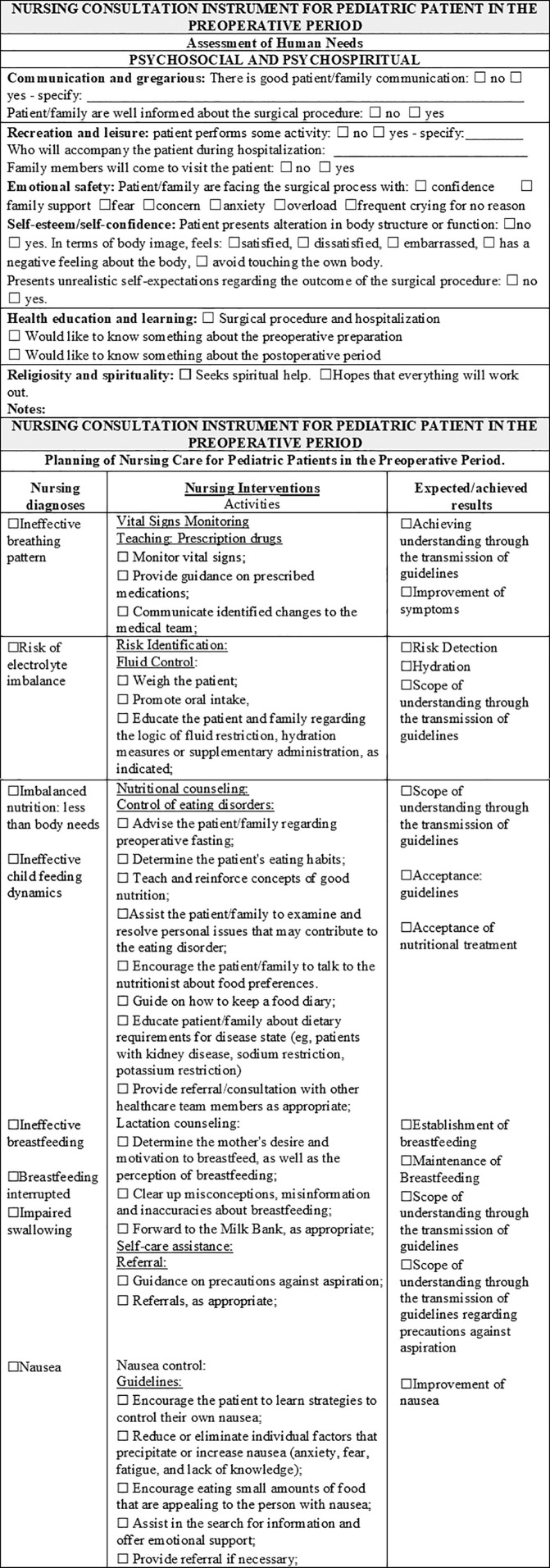
Final Version of the Instrument – Assessment of Human Needs: Psychosocial and Psychospiritual and Planning of Nursing Care to Pediatric Patients in Preoperative. Niterói, RJ, Brazil, 2020.

**Figure 3.**
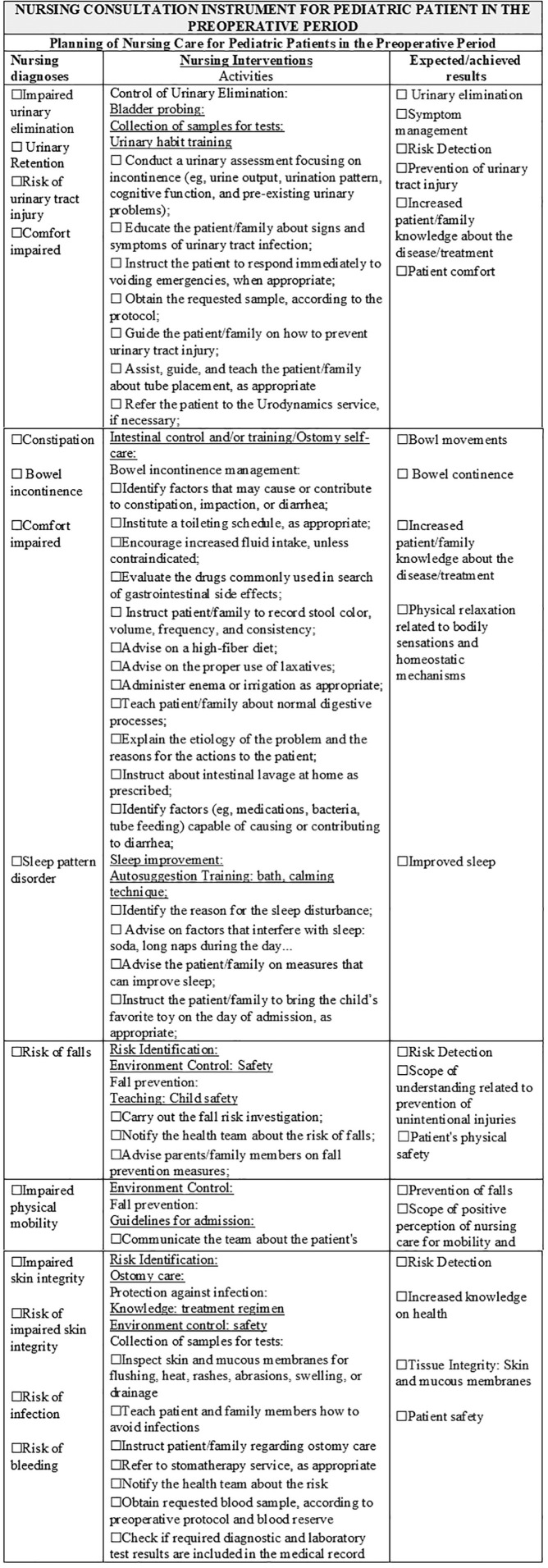
Final Version of the Instrument – Planning of Nursing Care to Pediatric Patients in the Preoperative Period. Niterói, RJ, Brazil, 2020.

**Figure 4.**
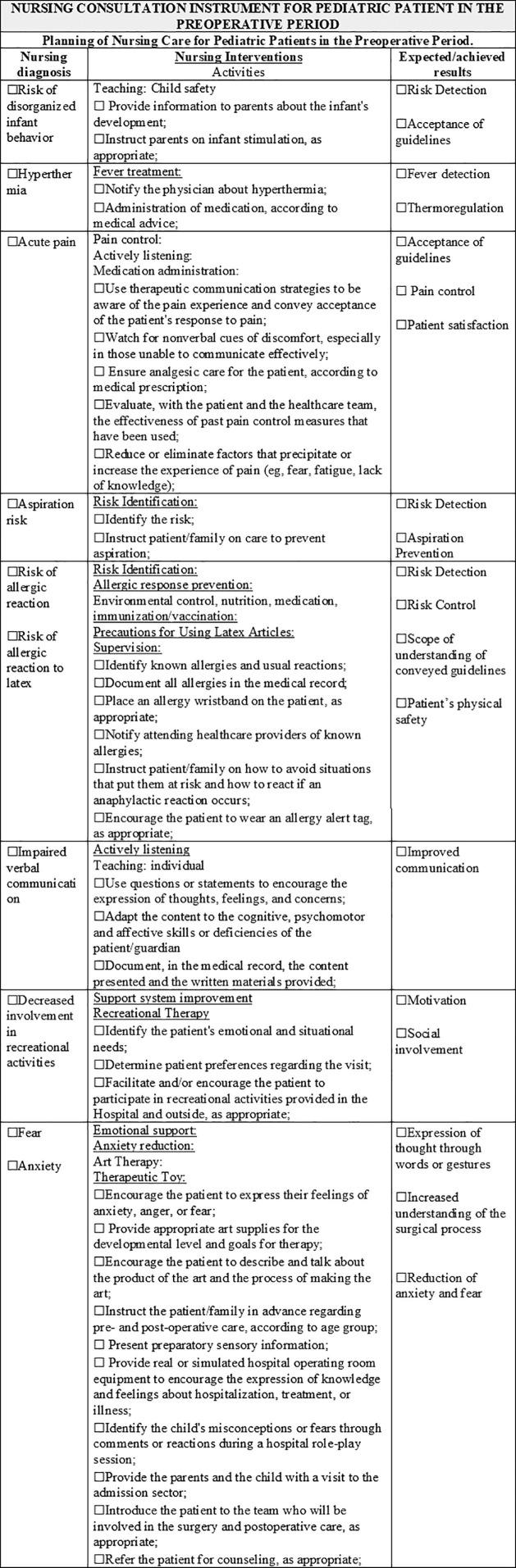
Final Version of the Instrument – Planning of Nursing Care for Pediatric Patients in the Preoperative Period. Niterói, RJ, Brazil, 2020.

**Figure 5.**
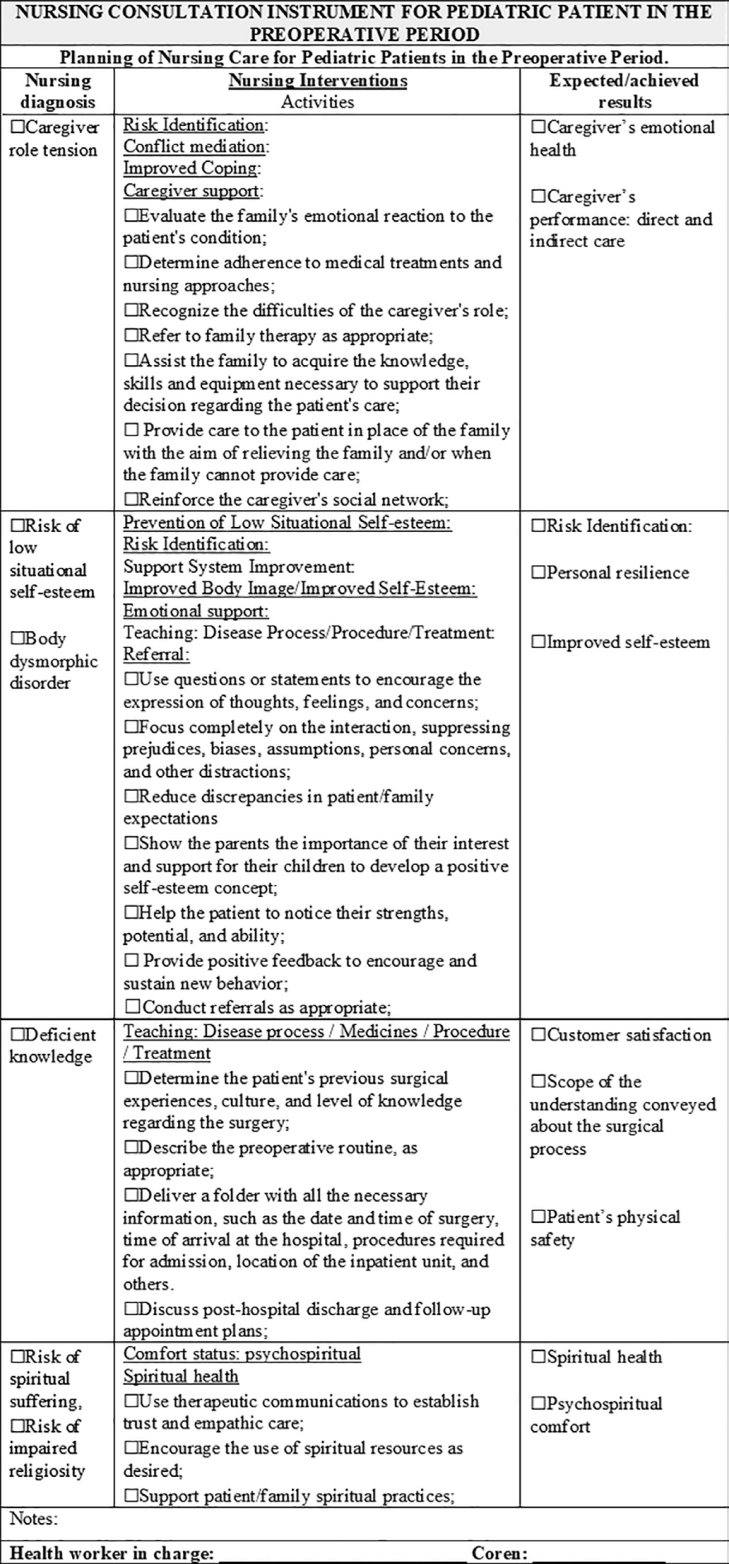
Final Version of the Instrument – Planning of Nursing Care for Pediatric Patients in the Preoperative Period. Niterói, RJ, Brazil, 2020.

Following the instrument validation process that considered the statements obtaining a CVI ≥ 0.70 as validated, the recommendations and suggestions of the specialists resulted in an instrument contemplating the assessment of psychobiological, psychosocial, and psychospiritual human needs, 38 nursing diagnoses, 65 nursing interventions, 113 nursing activities, and 62 nursing outcomes. The instrument obtained a content validity index (CVI) between 0.90 and 1.0 in the first round, and suggestions, validated in the second round, obtained agreement from 70 to 100%. As new rounds were not required, the validation of the instrument content was completed. In Figures 1 to 5, parts of the Instrument are illustrated.

## DISCUSSION

The performance of the NP is supported by public policies for children’s health care, especially with regard to the comprehensiveness of their care and individualized and resolute assistance^(18)^. Therefore, considering the NP as a qualification tool of care demonstrates the urgency of its development in the care of pediatric surgical patients^(19)^.

The selection of Wanda Horta’s Theory to guide the instrument construction and development is based on its significant importance for the development of the NP in Brazil, on its vision of how to assist the human being/patient, and on the possibility of identifying the legitimate needs expressed by the pediatric patient in the preoperative period, in addition to being the theory used by the nursing team in the institution where this study was carried out^(19,20)^.

Regarding the assessment of psychobiological needs, it should be noted that there are numerous symptoms and signs with potential risks to compromise the surgical pediatric patient safety and treatment. For similar reasons, psychosocial and psychospiritual needs can influence the entire course of treatment, and anxiety and lack of/or guidance offered not consistent with the needs of the patient and his/her family can lead to possible surgical postponement^(21)^.

During the Instrument development, the proximity with the clinical reality of the situations experienced by nurses, with the pediatric patient in the preoperative period, of various surgeries, was sought. The meetings held to identify the nursing diagnoses resulted in the 38 diagnoses used in the instrument, of which seven had been suggested by the investigator: impaired urinary elimination, urinary retention, risk of urinary tract injury, risk of allergic reaction to latex, body dysmorphic disorder, risk of spiritual suffering, and risk of impaired religiosity.

The inclusion of the diagnosis “impaired urinary elimination” is justified by the fact that it can be inferred by the nurse during the analysis of the child’s urinary elimination, in the outpatient consultation, and also because the accuracy of the identification of this diagnosis is of paramount importance to avoid medical complications for the patient^(22)^. The diagnosis of “urinary retention” has as defining characteristics the absence or reduction of urine elimination, as well as incontinence and bladder distention, common characteristics found in children with malformations treated at the study site^(12)^.

Therefore, the “risk of urinary tract injury” configures a possible diagnosis when these children need multiple catheterizations^(12)^. The risk is increased as some children, in addition to needing to undergo catheterization for months or years, have anatomical variations in pelvic organs. The patient should also be well evaluated regarding the diagnosis “risk of allergic reaction to latex”, as patients with histories of multiple surgical procedures and frequent exposure to products with latex may present with various symptoms, from the simplest, such as itching, to shock with circulatory collapse and cardiac arrest^(23,24)^.

When considering that the body image can be understood as an image that involves psychological, sociological, and physiological aspects that each individual forms of him/herself, NANDA ratifies the diagnosis “body dysmorphic disorder”, defining it as a “confusion in the mental image of the physical self”^(12)^. This diagnosis may be related to ostomized people or to some real change in body structure^(25)^.

It is understood that the diagnoses “risk of spiritual suffering” and “risk of impaired religiosity” are linked to the dimensions of the being that shall be considered in all fields of nursing care, as well as in research. However, as they are more subjective, they are still a challenge in care practice^(26)^.

The occurrence of NANDA-I diagnoses both in the online databases and in the dynamics with the institution’s nurses corroborates the thought that the development of the instrument with nursing diagnoses, interventions, and outcomes, aimed at the pediatric patient in the preoperative period, may contribute to the assistance provided and to document professional practice^(11)^.

The participation of nurses working in the institution where the instrument will be implemented was extremely relevant, as they provide assistance to the local clientele, know the routine of the unit, and are aware of what can be implemented. With regard to the nurses who participated in the validation of the instrument, it is important to highlight the magnitude of their collaboration through knowledge related to nursing care for the pediatric patient in question, ratifying the content used in the construction of the Instrument that was prepared and that will be used.

The limitation of this study is based on the scarcity of publications available in the literature that contemplate nursing care based on standardized nursing diagnoses and on the affected human needs of pediatric patients and their families in the surgical process. As a contribution to nursing, an instrument is made available aiming at a more effective care practice with the use of a tool that can guide systematic, safe and assertive nursing actions for this population.

## CONCLUSION

This study allowed content preparation and validation of the instrument for nursing consultations with pediatric patients in the preoperative period. Its use is important, since it standardizes and qualifies care, allowing the nurse to make a decision through clinical reasoning and considering the particularities of each surgical patient and their family.

The standardized languages of NANDA, NIC, and NOC proved to be adequate due to the range of possible nursing diagnoses, interventions, and outcomes for the pediatric patient in the preoperative period and for facilitating nursing care planning.

It is believed that the present study has the potential to stimulate new discussions about the safety of pediatric patients in the surgical process and regarding the use of a standardized clinical decision tool. The performance of future studies related to its application in the proposed scenario is recommended.

## ASSOCIATE EDITOR

Ivone Evangelista Cabral
